# Association between serum direct bilirubin and 90-day mortality in patients with ARDS: A retrospective analysis

**DOI:** 10.1097/MD.0000000000043051

**Published:** 2025-06-27

**Authors:** Jiao Xu, Jun Jin, Qing-Shan Zhou, Jiang-Tao Deng

**Affiliations:** a Department of Anesthesiology, The Eighth Affiliated Hospital of Sun Yat-sen University, Shenzhen, Guangdong, China; b Department of Critical Care Medicine, The University of Hong Kong-Shenzhen Hospital, Shenzhen, Guangdong, China.

**Keywords:** acute respiratory distress syndrome, direct bilirubin, mortality, prognostic marker, retrospective study

## Abstract

The liver plays a key role in the pathogenesis and resolution of acute respiratory distress syndrome (ARDS). Clinically, elevated serum bilirubin – especially direct bilirubin (DBIL) – is frequently observed in ARDS. This study aimed to evaluate the association between DBIL levels and 90-day mortality in ARDS patients. This retrospective cohort study used data from the MIMIC-IV database. ARDS patients were identified based on the Berlin definition. The primary outcome was 90-day all-cause mortality; in-hospital mortality was secondary. Cox proportional hazards models assessed the association between DBIL levels and mortality. Restricted cubic spline regression examined nonlinear relationships. Kaplan–Meier analysis compared survival across DBIL strata. A total of 714 ARDS patients were included. Patients with DBIL > 1.05 mg/dL had worse clinical profiles, including lower arterial pH, higher lactate, elevated ALT, and higher sequential organ failure assessment scores. Kaplan–Meier analysis showed significantly lower survival in the high DBIL group (52.2% vs 73.7%; *P* < .001). Multivariable Cox analysis showed elevated DBIL was independently associated with 90-day mortality (HR = 1.76; 95% CI = 1.33–2.33; *P* < .001) and in-hospital mortality (HR =1.99; 95% CI = 1.59–2.50; *P* < .001). Indirect bilirubin was not significantly associated with 90-day mortality. Restricted cubic spline analysis revealed a nonlinear relationship between DBIL and 90-day mortality (*P* for nonlinearity = .002). Our study demonstrates that DBIL is independently associated with 90-day mortality in patients with ARDS. Clinicians should consider close monitoring of DBIL levels and adjust management strategies accordingly to improve patient outcomes.

## 1. Introduction

Acute respiratory distress syndrome (ARDS) is characterized by acute hypoxemic respiratory failure and bilateral infiltrates on chest imaging that cannot be fully explained by cardiac failure or fluid overload.^[1]^ It is typically triggered by risk factors such as pneumonia, sepsis, aspiration, trauma or pancreatitis.^[2]^ The inciting injury initiates a cascade of inflammatory responses, disrupting alveolar-capillary barrier integrity. Pulmonary insults directly affect the alveolar epithelium, whereas extrapulmonary insults induce systemic inflammation and damage the vascular endothelium.^[3]^ ARDS affects approximately 10% of intensive care unit (ICU) patients globally and remains associated with high mortality, ranging from 30% to 40% in most studies.^[4,5]^ Consequently, reducing ARDS-related mortality is a critical and urgent priority.

The liver plays a central role in energy metabolism, detoxification, immune regulation, and systemic inflammation.^[6,7]^ Upon injury, cytokines such as interleukin-6 (IL-6) stimulate hepatic production of acute-phase proteins including C-reactive protein (CRP), fibrinogen, and serum amyloid A.^[8]^ Hepatic macrophages and mononuclear cells modulate immune responses and regulate systemic inflammation.^[1,9]^ Impaired hepatic clearance of endotoxins like lipopolysaccharide (LPS) may further exacerbate inflammatory responses.^[10]^ Evidence suggests that liver dysfunction significantly affects the pathophysiology and prognosis of ARDS.^[11]^ Serum bilirubin is widely used as an indicator of liver function. Elevated bilirubin levels, particularly direct bilirubin (DBIL), are commonly observed in ICU patients and have been associated with worse outcomes in sepsis and during extracorporeal membrane oxygenation (ECMO) support.^[12–14]^ While bilirubin possesses antioxidant properties under physiological conditions,^[15]^ excessive accumulation may promote oxidative stress, apoptosis, and inflammation.^[16]^ Recent studies have established bilirubin as a reliable predictor of disease severity and adverse clinical outcomes in critically ill patients.^[17]^ Studies in liver disease suggest that DBIL has superior prognostic value compared to total bilirubin (TBIL).^[18]^ However, the prognostic significance of DBIL in ARDS remains unclear.

Clinically, we have observed that ARDS patients with predominant DBIL elevation often experience repeated ICU admissions and poor long-term outcomes. Therefore, this study aims to investigate the association between DBIL levels and mortality outcomes – specifically 90-day and in-hospital mortality – in patients with ARDS. By elucidating this relationship, we aim to identify potential prognostic markers to inform risk stratification and guide individualized management in the ICU.

## 2. Materials and methods

### 2.1. Database and patients

The Medical Information Mart for Intensive Care-IV (MIMIC-IV) database is a publicly available resource with voluntary participation. Data collection and curation were approved by the Institutional Review Board of Beth Israel Deaconess Medical Center, which waived the requirement for individual informed consent and supported the data-sharing initiative. Therefore, no additional ethical approval was required for this study. The lead author, Jiang-Tao Deng, completed the required training provided by the U.S. National Institutes of Health and obtained authorized access to the database (Certificate No. 61424657).

This single-center retrospective cohort study included adult patients diagnosed with ARDS from the MIMIC-IV database. Jointly developed by the Massachusetts Institute of Technology, Beth Israel Deaconess Medical Center, and Philips Healthcare, MIMIC-IV contains detailed clinical data on over 40,000 ICU admissions – primarily of Caucasian patients – at Beth Israel Deaconess Medical Center between 2008 and 2019. The database is publicly accessible at https://physionet.org/content/mimiciv/2.2/, and all data used in this study were derived from it.

A total of 4405 ARDS patients were initially included in the study. Patients under 18 years of age and those lacking DBIL measurements following the diagnosis of ARDS were excluded. Patients were stratified into 2 groups based on DBIL levels (≤1.05 mg/dL or >1.05 mg/dL), using a predefined cutoff value. Patients were prospectively followed from ARDS diagnosis until the occurrence of either mortality or hospital discharge. The timing of ARDS diagnosis was defined as the point when the oxygenation index (OI) (PaO_2_/FiO_2_) first dropped to ≤ 300, in accordance with the Berlin definition of ARDS. Patients without DBIL measurements (n = 3691) and those under 18 years of age (n = 0) were excluded, leaving 714 patients who met the eligibility criteria. Bilirubin levels, along with other clinical indicators selected based on clinical experience and relevant literature, were collected for both 90-day survival and in-hospital survival analyses. The 90-day survival analysis covered the period from diagnosis until either 90 days post-diagnosis or death, whichever occurred first. In-hospital survival was assessed from diagnosis until either discharge or in-hospital mortality.

### 2.2. Data collection

Data were collected on a range of clinical and demographic variables, including age, gender, OI, partial pressure of carbon dioxide (PCO_2_), pH, base excess (BE), lactate, direct bilirubin (DBIL), total bilirubin (TBIL), indirect bilirubin (IBIL), alanine aminotransferase (ALT), white blood cell count (WBC), mean arterial pressure (MAP), creatinine, hemoglobin, international normalized ratio (INR), and positive end-expiratory pressure (PEEP). All of these variables were recorded based on their first measured values following the diagnosis of ARDS. The 24-hour sequential organ failure assessment (SOFA) score and the 24-hour acute physiology and chronic health evaluation II (APACHE II) score were also recorded. Additionally, data on norepinephrine infusion rate, survival outcomes, marital status (divorced, married, single, or other), and comorbidities including diabetes, hypertension, and heart failure were collected.

### 2.3. Primary outcome

The primary outcome was 90-day all-cause mortality, with patients surviving beyond this period or discharged alive classified as survivors. In-hospital mortality served as the secondary outcome.

### 2.4. Statistical analysis

All statistical analyses were conducted using R software (version 4.4.1; R Foundation for Statistical Computing, Vienna, Austria). A 2-sided *P*-value < .05 was considered statistically significant.

Continuous variables were expressed as mean ± standard deviation and compared using the Student *t* test. Categorical variables were presented as counts (percentages) and compared using the chi-square test. ROC curve analysis was used to determine the optimal cutoff for serum DBIL. Survival was analyzed using Kaplan–Meier (KM) curves and the log-rank test.

Variables with variance inflation factor > 5 (e.g., pH, BE) were excluded. The proportional hazards assumption was tested using Schoenfeld residuals (violated by PEEP, diabetes, and hemoglobin). Cox proportional hazards models were used to assess the association between DBIL and mortality. Three models were applied: Model 1 (unadjusted), Model 2 (adjusted for age and gender), and Model 3 (further adjusted for DBIL, APACHE II, SOFA, MAP, comorbidities, lab parameters, and norepinephrine dose).

Hazard ratios (HRs) and 95% confidence intervals were reported for both continuous and categorical DBIL (≤1.05 vs >1.05 mg/dL). Trend tests assessed linear associations. Restricted cubic spline (RCS) analysis was performed to evaluate nonlinear relationships.

Subgroup analyses were conducted based on gender, age (>65 vs ≤65), lactate (>2 vs ≤2 mmol/L), SOFA (>8 vs ≤8), APACHE II (>15 vs ≤15), diabetes, heart failure, and hypertension. Interaction terms were used to test for effect modification.

## 3. Results

### 3.1. Characteristics of patients with ARDS

**Figure 1.**
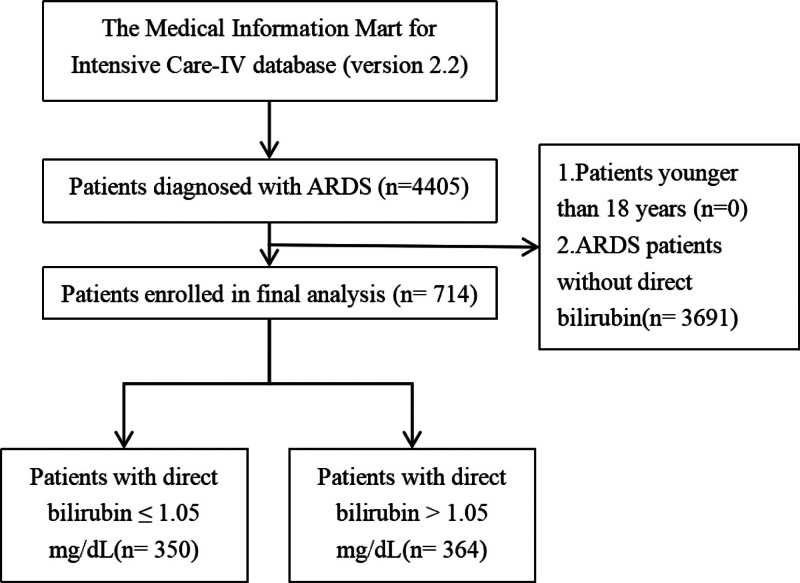
Flow diagram illustrating the screening process for the study population.

The screening process for the study population is illustrated in Figure 1. A total of 4405 patients diagnosed with ARDS were identified from the MIMIC-IV database (version 2.2). After applying exclusion criteria, 3691 patients were excluded due to the absence of DBIL measurements, and no patients were excluded for being younger than 18 years old. Ultimately, 714 patients were included in the final analysis.

These patients were further divided into 2 groups based on their baseline DBIL levels: 350 patients with DBIL levels ≤ 1.05 mg/dL and 364 patients with DBIL levels > 1.05 mg/dL.

Table 1 presents the baseline characteristics of ARDS patients stratified by DBIL levels. Patients in the lower DBIL group were significantly older than those in the higher group (60.93 ± 14.28 vs 58.16 ± 14.54 years; *P* = .011). No significant difference in gender distribution was observed between the 2 groups (*P* = .457).

**Table 1:** Characteristics of ARDS patients by direct bilirubin levels.

Variables	Lower DBIL group (n = 350)	Higher DBIL group (n = 364)	*P*-value
Age, yr	60.93 ± 14.28	58.16 ± 14.54	.011
Gender, n (%)			
Female	150 (42.9)	145 (39.8)	.457
Male	200 (57.1)	219 (60.2)	
Oxygenation index (mm Hg)	174.98 ± 72.67	178.00 ± 74.88	.585
PCO_2_ (mm Hg)	41.73 ± 9.45	40.43 ± 8.98	.060
Ph	7.36 ± 0.09	7.34 ± 0.10	.017
Base excess (mmol/L)	-1.43 ± 5.16	-2.89 ± 5.18	<.001
Lactate (mmol/L)	2.28 ± 1.58	3.12 ± 2.32	<.001
Direct bilirubin (mg/dL)	0.39 ± 0.28	3.85 ± 2.35	<.001
Total bilirubin (mg/dL)	1.29 ± 2.19	2.57 ± 3.47	<.001
Indirect bilirubin (mg/dL)	0.71 ± 0.83	1.87 ± 1.51	<.001
ALT (U/L)	112.78 ± 262.47	175.67 ± 357.48	.008
MAP (mm Hg)	71.57 ± 15.15	69.78 ± 14.99	.113
Creatinine (mg/dL)	1.48 ± 1.21	1.79 ± 1.24	.001
Hemoglobin (g/dL)	9.63 ± 1.95	9.34 ± 1.92	.042
INR	1.52 ± 0.66	1.74 ± 0.72	<.001
PEEP	6.71 ± 2.72	6.75 ± 2.92	.845
24-h SOFA score	6.83 ± 3.97	9.87 ± 3.99	<.001
24-h APACHE II score	18.07 ± 6.07	19.15 ± 6.00	.018
WBC (×10^9^/L)	12.44 ± 11.81	13.51 ± 11.54	.220
Norepinephrine rate (μg/kg/min)	0.16 ± 0.10	0.18 ± 0.13	.025
Survival, n (%)			
Yes	258 (73.7)	190 (52.2)	<.001
No	92 (26.3)	174 (47.8)	
Marital status, n (%)			
Divorced	38 (10.9)	34 (9.3)	.119
Married	160 (45.7)	135 (37.1)	
Single	8 (2.3)	24 (6.6)	
Other	144 (41.2)	171 (47.0)	
Diabetes, n (%)			
Yes	200 (57.1)	227 (62.4)	.178
No	150 (42.9)	137 (37.6)	
Hypertension, n (%)			
Yes	90 (25.7)	99 (27.2)	.716
No	260 (74.3)	265 (72.8)	
Heart failure, n (%)			
Yes	192 (54.9)	244 (67.0)	.001
No	158 (45.1)	120 (33.0)	

ALT = alanine aminotransferase, APACHE II = acute physiology and chronic health evaluation II, ARDS = acute respiratory distress syndrome, DBIL = direct bilirubin, INR = international normalized ratio, MAP = mean arterial pressure, PCO_2_ = pressure of carbon dioxide, PEEP = and positive end-expiratory pressure, SOFA = sequential organ failure assessment, WBC = white blood cell count.

There was no significant difference in the OI between the 2 groups (*P*-value = .585). However, patients in the higher DBIL group had a significantly lower arterial pH (7.36 ± 0.09 vs 7.34 ± 0.10; *P*-value = .017) and a more negative BE (−1.43 ± 5.16 vs −2.89 ± 5.18 mmol/L; *P*-value < .001). Serum lactate levels were also markedly higher in the higher DBIL group (2.28 ± 1.58 vs 3.12 ± 2.32 mmol/L; *P*-value < .001).

As expected, levels of DBIL (0.39 ± 0.28 vs 3.85 ± 2.35 mg/dL; *P*-value < .001), total bilirubin (TBIL) (1.29 ± 2.19 vs 2.57 ± 3.47 mg/dL; *P*-value < .001), and indirect bilirubin (IBIL) (0.71 ± 0.83 vs 1.87 ± 1.51 mg/dL; *P*-value < .001) were significantly elevated in the higher group. Alanine aminotransferase (ALT) levels were also significantly higher in this group (112.78 ± 262.47 vs 175.67 ± 357.48 U/L; *P*-value = .008). Likewise, hemoglobin (HGB) levels were slightly but significantly lower in the higher group (9.63 ± 1.95 vs 9.34 ± 1.92 g/dL; *P*-value = .042).

MAP did not differ significantly between the groups (*P*-value = .113). However, the INR was significantly higher in the higher DBIL group (1.52 ± 0.66 vs 1.74 ± 0.72; *P*-value < .001). Both the 24-hour SOFA score and the 24-hour acute physiology and chronic health evaluation II (APACHE II) score were significantly elevated in the higher DBIL group (*P*-value < .05). Notably, survival rates were significantly lower in the higher DBIL group compared to the lower group (52.2% vs 73.7%; *P*-value < .001).

The prevalence of diabetes mellitus and hypertension did not differ significantly between the groups (*P*-value = .178 and *P*-value = .716, respectively). However, the proportion of patients with heart failure was significantly higher in the higher DBIL group (67.0% vs 54.9%; *P*-value = .001).

### 3.2. Kaplan–Meier survival analysis by direct bilirubin levels in ARDS patients

**Figure 2.**
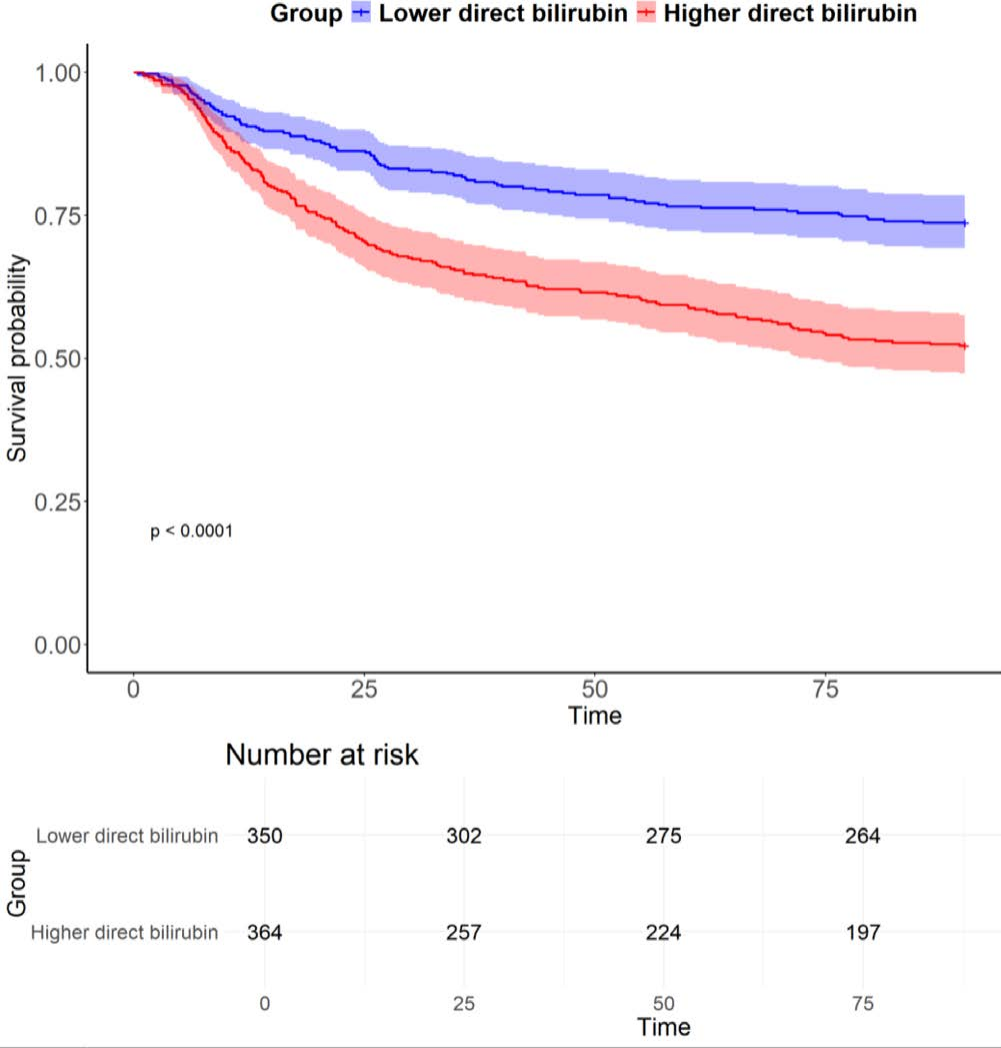
Kaplan–Meier survival analysis by levels. DBIL = direct bilirubin.

The KM survival analysis comparing ARDS patients with lower and higher DBIL levels is shown in Figure 2. Patients in the higher DBIL group exhibited significantly reduced survival probabilities compared to those in the lower DBIL group (*P* < .001). The survival curves diverged early during the follow-up period and continued to separate over time, indicating a persistent survival disadvantage associated with elevated DBIL levels.

The numbers at risk at each time point are also presented. By the end of the follow-up period, the number at risk declined to 264 in the lower DBIL group and 197 in the higher DBIL group.

### 3.3. Association between direct bilirubin levels and mortality risk based on restricted cubic spline analysis

**Figure 3.**
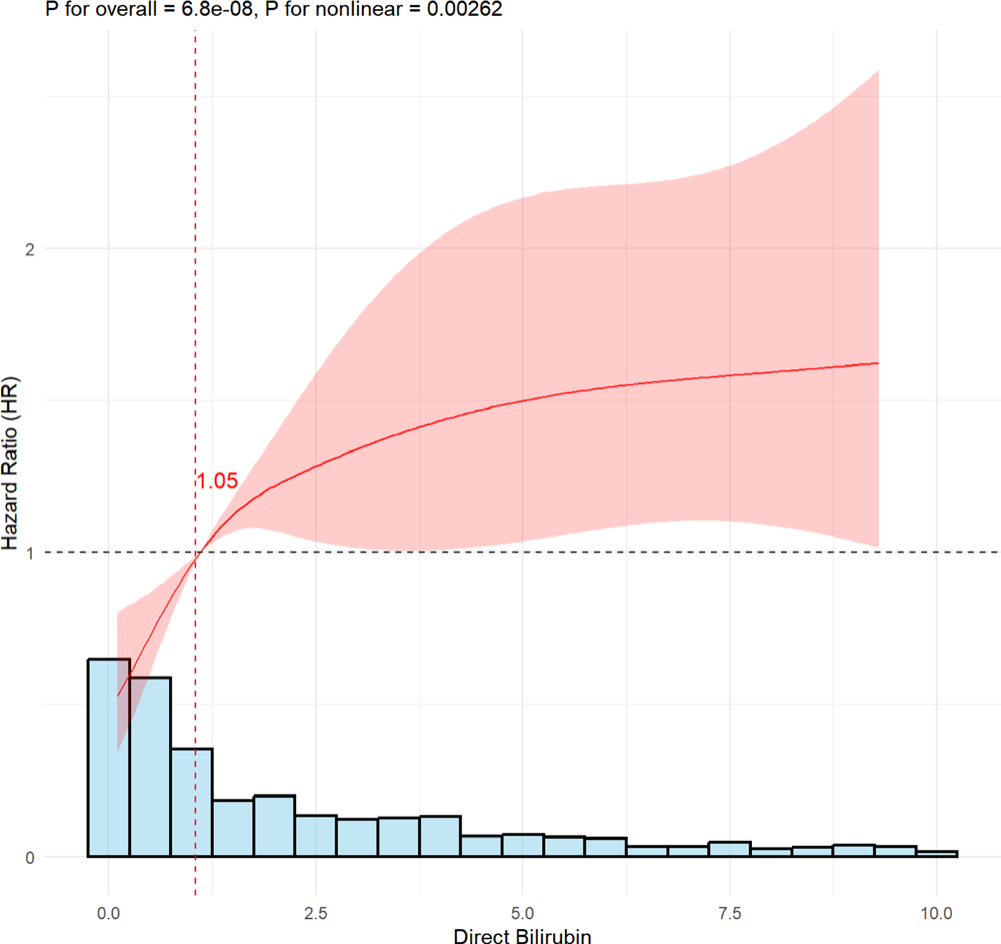
Restricted cubic spline analysis of DBIL and 90-d mortality. DBIL = direct bilirubin.

Figure 3 presents the results of the RCS analysis evaluating the association between DBIL levels and the HR for 90-day mortality. The curve reveals a steep initial increase in HR with rising DBIL levels, which subsequently transitions to a more gradual upward trend at higher concentrations. The reference value was set at 1.05 mg/dL (denoted by the vertical dashed red line), corresponding to an HR of 1.00, indicating no change in mortality risk at this level.

The overall association between DBIL and mortality was statistically significant (*P* for overall < .001), as was the nonlinear relationship (*P* for nonlinearity = .002). The curve’s configuration supports a nonlinear association, characterized by a sharp rise in risk at lower DBIL levels that attenuates as DBIL increases.

### 3.4. Association between direct bilirubin levels and mortality outcomes in ARDS

A Schoenfeld residual test was performed to assess the proportional hazards assumption. Variables including PEEP, diabetes, and HGB violated this assumption significantly (*P* < .05) and were therefore excluded from the final Cox regression model.

Table 2 summarizes the results of Cox proportional hazards regression analyses examining the relationship between DBIL levels and mortality outcomes (90-day and in-hospital mortality) in ARDS patients. Three models were used: Model 1 (unadjusted), Model 2 (adjusted for age and gender), and Model 3 (fully adjusted for multiple covariates, including gender, age, IBIL, WBC, MAP, 24-hour SOFA score, heart failure, hypertension, OI, ALT, creatinine, lactate, PCO_2_, and norepinephrine infusion rate).

**Table 2:** Cox proportional hazards regression analyses of direct bilirubin and 90-d and in-hospital mortality in patients with ARDS.

Variables	Model 1	*P*-value	Model 2	*P*-value	Model 3	*P*-value
	HR (95% CI)		HR (95% CI)		HR (95% CI)	
90-d mortality						
Per 1-unit increase	1.12 (1.08–1.17)	<.001	1.14 (1.09–1.19)	<.001	1.11 (1.05–1.16)	<.001
Lower direct bilirubin	1 (Reference)	–	1 (Reference)	–	1 (Reference)	–
Higher direct bilirubin	2.11 (1.64–2.72)	<.001	2.18 (1.69–2.81)	<.001	1.76 (1.33–2.33)	<.001
*P* for trend	<.001	–	<.001	–	<.001	–
In-hospital mortality						
Per 1-unit increase	1.14 (1.10–1.19)	<.001	1.16 (1.12–1.21)	<.001	1.14 (1.09–1.18)	<.001
Lower direct bilirubin	1 (Reference)	–	1 (Reference)	–	1 (Reference)	–
Higher direct bilirubin	2.06 (1.68–2.53)	<.001	2.25 (1.83–2.76)	<.001	1.99 (1.59–2.50)	<.001
*P* for trend	<.001	–	<.001	–	<.001	–

Model 1: unadjusted. Model 2: adjusted for age and gender. Model 3: adjusted for gender, age, indirect bilirubin, 24-hour APACHE II score, MAP, 24-hour SOFA score, heart failure, hypertension, WBC, OI, ALT, creatinine, lactate, partial PCO_2_, and norepinephrine infusion rate.

ALT = alanine aminotransferase, APACHE II = acute physiology and chronic health evaluation II, ARDS = acute respiratory distress syndrome, CI = confidence interval, HR = hazard ratio, MAP = mean arterial pressure, OI = oxygenation index, PCO_2_ = pressure of carbon dioxide, SOFA = sequential organ failure assessment, WBC = white blood cell count.

For 90-day mortality, each 1-unit increase in DBIL was associated with a higher HR in all models. In the fully adjusted Model 3, the HR was 1.11 (95% CI = 1.05–1.16, *P* < .001). Compared to the lower DBIL group, the higher DBIL group demonstrated a significantly increased risk of 90-day mortality, with an HR of 1.76 (95% CI = 1.33–2.33, *P* < .001). A significant dose-response trend was observed across bilirubin levels (*P* for trend < .001).

For in-hospital mortality, similar trends were observed. Each 1-unit increase in DBIL was associated with an increased risk of mortality, with an HR of 1.14 (95% CI = 1.09–1.18, *P* < .001) in Model 3. The higher DBIL group had a significantly higher risk of in-hospital mortality compared to the lower DBIL group, with an HR of 1.99 (95% CI = 1.59–2.50, *P* < .001). The *P* for trend was also significant across all models (*P* for trend < .001).

### 3.5. Association of direct and IBIL levels with 90-day mortality in ARDS patients: cox proportional hazards regression analysis

Cox proportional hazards regression analysis showed that DBIL was significantly associated with 90-day mortality in ARDS patients. In the unadjusted model (Model 1), the HR for DBIL was 1.12 (95% CI = 1.08–1.17, *P* < .001). This association remained significant after adjusting for age and gender (Model 2: HR 1.14, 95% CI = 1.09–1.19, *P* < .001) and further adjustment for clinical and laboratory variables (Model 3: HR 1.10, 95% CI = 1.05–1.16, *P* < .001).

In contrast, IBIL was not significantly associated with 90-day mortality in any model. In Model 1, the HR was 1.06 (95% CI = 0.97–1.15, *P* = .208), and in the fully adjusted Model 3, the HR was 1.02 (95% CI = 0.92–1.13, *P* = .711).

### 3.6. Subgroup analyses

**Figure 4.**
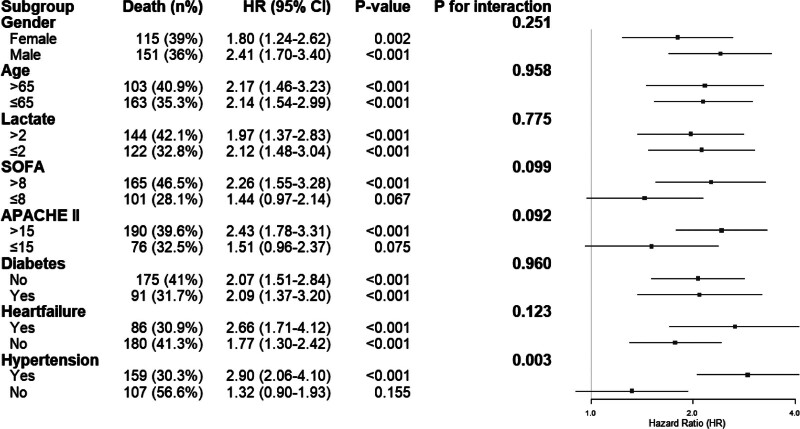
Subgroup analyses of the association between DBIL and clinical outcomes. DBIL = direct bilirubin.

Figure 4 presents the results of subgroup analyses evaluating the association between DBIL levels and 90-day mortality in patients with ARDS. Overall, elevated DBIL was significantly associated with an increased risk of mortality across most clinical subgroups.

The strength of the association was consistent across gender (females: HR = 1.80, 95% CI = 1.24–2.62; males: HR = 2.41, 95% CI = 1.70–3.40; *P* for interaction = .251) and age groups (>65 years: HR = 2.17, 95% CI = 1.46–3.23; ≤65 years: HR = 2.14, 95% CI = 1.54–2.99; *P* for interaction = .958). Similarly, no significant effect modification was observed with respect to serum lactate, SOFA score, APACHE II score, diabetes, or heart failure (all *P* for interaction > .05).

Notably, a statistically significant interaction was observed for hypertension status (*P* for interaction = .003). Among patients without hypertension, DBIL was strongly associated with mortality (HR = 2.90, 95% CI = 2.06–4.10), whereas the association was not significant in those with hypertension (HR = 1.32, 95% CI = 0.90–1.93). These findings suggest that the prognostic impact of DBIL may be influenced by baseline hypertension status.

## 4. Discussion

In this study, elevated serum DBIL levels measured after ARDS diagnosis were significantly associated with increased 90-day all-cause mortality, even after adjusting for potential confounders. In contrast, no such association was observed for indirect bilirubin (IBIL). This association remained consistent across most clinical subgroups. Furthermore, higher DBIL levels were linked to increased in-hospital mortality, suggesting that DBIL may serve as an independent prognostic biomarker and a valuable parameter for risk stratification in ARDS patients.

Liver dysfunction is a key determinant of mortality in ARDS patients.^[19]^ Growing evidence indicates that liver injury exacerbates pulmonary inflammation and disrupts lung structure and function.^[20]^ Impairment of the hepatic reticuloendothelial system permits bacterial products to enter the systemic circulation and lungs, triggering both systemic and pulmonary inflammation.^[21]^ The liver is essential for protein synthesis, toxin metabolism, and regulation of systemic inflammation and host defense. Preserved liver function appears to protect against lung injury, underscoring its role in recovery.^[22]^ In summary, the liver is a critical modulator of systemic inflammation and ARDS.

Total bilirubin (TBIL) is a routinely measured biomarker in primary care, primarily used to diagnose hepatobiliary and hemolytic disorders.^[23]^ Sheu et al demonstrated that elevated TBIL levels at ICU admission were significantly associated with 60-day mortality in ARDS patients.^[24]^ Emerging evidence suggests that DBIL may be a more robust prognostic indicator than TBIL in patients with liver cirrhosis. Additionally, a large cohort study involving 19,837 patients reported that DBIL – but not IBIL – was associated with poor short-term outcomes in heart failure with preserved ejection fraction (HFpEF), further supporting its prognostic value.^[25]^

In this study, a total of 4405 ARDS patients, identified according to the Berlin Definition, were retrieved from the MIMIC-IV database. Of these, 714 patients met the inclusion criteria and were stratified into 2 groups based on a DBIL threshold of 1.05 mg/dL. Alanine aminotransferase (ALT) levels were significantly higher in the elevated DBIL group, supporting the utility of DBIL as a surrogate marker of liver function. Furthermore, a significant difference in the incidence of heart failure was observed between the 2 groups, suggesting a potential role of hepatic congestion in DBIL elevation. KM survival analysis (Fig. 2) demonstrated that patients with higher DBIL levels had significantly worse survival outcomes compared to those with lower levels. These findings align with previous studies reporting an association between elevated DBIL and adverse prognosis, such as in a cohort of 337 critically ill COVID-19 patients.^[26]^ These findings suggest that elevated DBIL levels may serve as a prognostic marker in ARDS patients, underscoring the value of DBIL monitoring in identifying individuals at increased mortality risk. To further explore this association, RCS analysis was performed to examine the relationship between DBIL levels and 90-day mortality. As shown in Figure 3, the HR for mortality increased significantly when DBIL levels exceeded 1.05 mg/dL (*P* for overall < .001; *P* for nonlinearity = .002), indicating a nonlinear association between DBIL and mortality risk. Higher DBIL levels were correlated with progressively worse outcomes. Consistent with our results, previous studies have also reported a nonlinear relationship between TBIL levels and outcomes in ARDS patients,^[27]^ this association may be primarily driven by the effects of DBIL. Cox proportional hazards regression analysis (Table 2) demonstrated a significant association between DBIL levels and both 90-day and in-hospital mortality in ARDS patients. Across all 3 models – unadjusted (Model 1), adjusted for age and sex (Model 2), and fully adjusted for clinical and biochemical variables (Model 3) – elevated DBIL levels were consistently associated with increased HRs for mortality. In the fully adjusted model, the HR for 90-day mortality in the high DBIL group was 1.76 (95% CI = 1.33–2.33; *P* < .001). To account for potential survival bias due to incomplete follow-up, we also examined the relationship between TBIL and in-hospital mortality. Similarly, the HR for in-hospital mortality in Model 3 was 1.99 (95% CI = 1.59–2.50; *P* < .001). These results underscore a robust association between elevated DBIL levels and poor outcomes, reinforcing the prognostic utility of DBIL in ARDS. The consistent trend across all models highlights the independent contribution of DBIL to mortality risk.

What might explain this association between elevated DBIL and 90-day mortality in ARDS? Two key mechanisms may underlie this relationship. First, DBIL is a well-established marker of liver dysfunction, which may reflect the extent of liver injury caused by ARDS. In ARDS, hypoxia and systemic inflammation – mediated by cytokines and endotoxins – can induce microcirculatory disturbances and hepatic injury, potentially exacerbating liver dysfunction and contributing to worse outcomes.^[28]^ These disturbances may lead to edema and injury of the biliary canaliculi, impairing bile excretion and resulting in elevated DBIL levels. Post-coronavirus disease 2019 (COVID-19), which is classified as a form of ARDS, cholangiopathy has emerged as one of the most severe long-term gastrointestinal complications.^[29]^ Cholestasis is a common complication in sepsis patients, primarily driven by an exaggerated inflammatory response.^[30]^ Experimental evidence from animal models of sepsis shows that proinflammatory cytokines and mediators downregulate key hepatocellular transporters, thereby linking systemic inflammation to hepatocellular cholestasis.^[31]^ Similarly, ARDS may exacerbate the proinflammatory state, leading to hepatic injury and elevated DBIL levels. In summary, elevated DBIL may reflect not only hepatic injury but also uncontrolled systemic inflammation and infection induced by ARDS. Therefore, higher DBIL levels may indicate more severe disease progression in ARDS, ultimately contributing to increased 90-day mortality. The data in Table 1 show no significant differences in OI, arterial partial pressure of carbon dioxide (PaCO_2_), or MAP between the 2 groups, suggesting that ischemia and hypoxia are unlikely to be major contributors to liver dysfunction. Second, impaired hepatic function may prolong and complicate the clinical course of ARDS. Guillot et al emphasized that the hepatobiliary system plays a pivotal role in detoxifying proinflammatory cytokines, vasoactive mediators, and eicosanoids from the systemic circulation. Efficient clearance of these mediators is essential for maintaining systemic and pulmonary immune homeostasis, thereby protecting the lungs and other extrahepatic organs from injury. As noted by the authors, hepatic injury exacerbates systemic inflammation, potentially contributing to disease progression and poor outcomes.^[32]^ Wang et al further demonstrated that hepatic mononuclear cells – including lymphocytes, Kupffer cells, monocytes, and granulocytes – are essential components of both innate and adaptive immunity. During hepatic injury, activation of these cells leads to increased production and systemic release of proinflammatory mediators, such as interleukin (IL)-1, IL-6, tumor necrosis factor-alpha (TNF-α), platelet-activating factor, and leukotrienes. The authors emphasized that these mediators play a central role in lung–liver crosstalk, modulating inflammatory responses and inter-organ signaling.^[33]^ Table 1 also shows that ALT and INR levels are higher in the high DBIL group, indicating worse liver function. However, due to the limitations of the MIMIC database, sufficient data on inflammatory markers such as IL-1, IL-6, and TNF-α are unavailable, preventing assessment of the inflammatory status in this group and its potential impact on ARDS.

The Cox proportional hazards regression analysis in Table 3 shows the association between direct and IBIL levels and 90-day mortality in ARDS patients. DBIL levels were significantly associated with 90-day mortality across all 3 models. In Model 1 (unadjusted), the HR for DBIL was 1.12 (95% CI =1.08–1.17, *P* < .001). After adjusting for age and gender in Model 2, the HR remained 1.14 (95% CI = 1.09–1.20, *P* < .001). In the fully adjusted Model 3, which included 24-hour SOFA score, 24-hour APACHE II score, MAP, comorbidities, and laboratory parameters, the HR for DBIL was 1.10 (95% CI =1.05–1.16, *P* = .001). These findings highlight the distinct roles of direct and IBIL in predicting 90-day mortality in ARDS patients.

**Table 3:** Association of direct and indirect bilirubin levels with 90-d mortality in ARDS patients: cox proportional hazards regression analysis.

Variables	Model 1	*P*-value	Model 2	*P*-value	Model 3	*P*-value
	HR (95% CI)		HR (95% CI)		HR (95% CI)	
Direct bilirubin	1.12 (1.08–1.17)	<.001	1.14 (1.09–1.19)	<.001	1.10 (1.05–1.16)	<.001
Indirect bilirubin	1.06 (0.97–1.15)	.208	1.07 (0.98–1.17)	.124	1.02 (0.92–1.13)	.711

Model 1: unadjusted. Model 2: adjusted for age and gender. Model 3: adjusted for gender, age, indirect bilirubin, 24-hour APACHE II score, MAP, 24-hour SOFA score, heart failure, hypertension, WBC, OI, ALT, creatinine, lactate, partial PCO_2_, and norepinephrine infusion rate.

ALT = alanine aminotransferase, APACHE II = acute physiology and chronic health evaluation II, ARDS = acute respiratory distress syndrome, CI = confidence interval, HR = hazard ratio, MAP = mean arterial pressure, OI = oxygenation index, PCO_2_ = pressure of carbon dioxide, SOFA = sequential organ failure assessment, WBC = white blood cell count.

Figure 4 shows subgroup analyses of the association between DBIL levels and 90-day mortality in ARDS patients. Elevated DBIL was significantly associated with higher mortality across most subgroups. However, a significant interaction with hypertension was observed (*P* for interaction = .003). Among patients without hypertension, elevated DBIL markedly increased mortality risk, while this association was not significant in those with hypertension. One possible explanation for this discrepancy is the common use of antihypertensive drugs – such as diuretics, β-blockers, and angiotensin-converting enzyme inhibitors – in patients with hypertension. Prehospital use of angiotensin-converting enzyme inhibitors or angiotensin receptor blockers has been associated with better outcomes in patients with acute respiratory failure and may serve as an independent prognostic factor.^[34]^ In patients with ARDS, early diuretic use was independently linked to reduced hospital mortality.^[35]^ Similarly, β-blockers have been associated with lower mortality in critically ill ARDS patients.^[36]^

Bilirubin is a metabolic byproduct of hemoglobin,^[37]^ which is readily accessible in clinical practice. DBIL holds significant clinical value in assessing the prognosis of ARDS, as its elevation is often indicative of hepatic dysfunction, systemic inflammation, or organ failure. In clinical practice, persistent or predominantly elevated DBIL should prompt a thorough evaluation to ensure that the underlying infection is adequately controlled, to investigate the potential presence of occult infections, to confirm that the primary cause of ARDS has been addressed, and to assess the management of systemic inflammation. Furthermore, the possibility of missed or overlooked diagnoses should be carefully considered. These evaluations are crucial for determining the effectiveness of ARDS treatment and for improving patient outcomes.

This study has several limitations. First, its retrospective design may introduce inherent biases, such as selection bias and confounding, potentially affecting the observed association between DBIL levels and mortality. Moreover, causality cannot be established due to the observational nature of the study. Second, only serum DBIL levels were analyzed, which may not fully reflect hepatic function or systemic inflammation. Inflammatory markers such as IL-6 and TNF-α were unavailable in the MIMIC-IV database, limiting further analysis. Third, the underlying mechanisms linking elevated DBIL to ARDS prognosis remain unclear and warrant further investigation. Fourth, differences in known and unknown confounders across bilirubin subgroups may have influenced the results. Although multivariable adjustments were performed, residual confounding cannot be ruled out. Fifth, being a single-center study based on the MIMIC-IV database, external validation is needed to enhance generalizability. Lastly, the absence of dynamic DBIL measurements during ICU stay limited the evaluation of bilirubin trends and their prognostic implications. Therefore, these findings should be interpreted with caution. Future prospective studies with serial bilirubin monitoring are needed to validate our results and explore potential mechanisms.

## 5. Conclusion

In conclusion, our study demonstrates that elevated DBIL levels are significantly associated with increased 90-day mortality in ARDS patients, whereas no such association was observed with indirect bilirubin (IBIL).

## Acknowledgments

We thank all participants and investigator involved in MIMIC-IV database for sharing data. Thanks to all the workers contributed to this study.

## Author contributions

**Conceptualization:** Jiao Xu.

**Validation:** Jun Jin.

**Writing – review & editing:** Qing-Shan Zhou.

**Writing – original draft:** Jiang-Tao Deng.
